# Liberal surgical laparoscopy reduction for acute intussusception: experience from a tertiary pediatric institute

**DOI:** 10.1038/s41598-023-50493-7

**Published:** 2024-01-03

**Authors:** Jian Yang, Guoyong Wang, Jia Gao, Xiaotong Zhong, Kai Gao, Qianyang Liu, Guoxin Nan, Chengwei Yan, Gongli Chen, Peng Lu, Chunbao Guo

**Affiliations:** 1 Department of Pediatric General Surgery, Yongchuan Maternal and Child Health Hospital, Chongqing, People’s Republic of China; 2 Department of Pediatric General Surgery, Chongqing Health Center for Women and Children, Chongqing Medical University, Chongqing, People’s Republic of China; 3 Department of Pediatrics, Women’s and Children’s Hospital, Chongqing Medical University, 120 Longshan Rd, Chongqing, 401147 People’s Republic of China; 4 Department of Pediatrics, Hechuan Maternal and Child Health Hospital, Chongqing, People’s Republic of China; 5 Department of Pediatrics, Three Gorges Hospital, Chongqing University, Chongqing, People’s Republic of China

**Keywords:** Diseases, Gastroenterology, Medical research

## Abstract

The optimal treatment for acute intussusception has not yet been defined. In this study, we explored whether employing a liberal laparoscopic intervention for intussusception could lead to favorable outcomes. We performed a historical control analysis to evaluate the outcomes associated with this liberal surgical management protocol. This liberal surgical management protocol were revised to incorporate a new protocol centered around the laparoscopic approach. In some cases of acute intussusception, liberal laparoscopic exploration and intervention were undertaken without initial hydrostatic or pneumatic reduction. During the study interval, a retrospective review was conducted on a total of 3086 patients. These were categorized into two groups: 1338 cases before May 2019 (pre-protocol group) and 1748 cases after May 2019 (post-protocol group). Surgical intervention rates in the pre-protoco and post-protocol period were 10.2% and 27.4% respectively (odds ratio [OR] = 0.30 [95% CI 0.25–0.37]; p < 0.001). No significant differences were observed in baseline clinical characteristics or demographic features between the two groups. The duration from admission to operation was longer for the pre-protocol group (p = 0.008) than for the post-protocol group. The post-protocol group demonstrated decreases in both intestinal resection (OR = 1.50 [95% CI 0.96–2.35]; p = 0.048) and total recurrent events (OR = 1.27 [95% CI 1.04–1.55]; p = 0.012) compared to the pre-protocol group. Liberal laparoscopic intervention for intussusception may effectively reduce the risk of intestinal resection and total recurrent events, thereby exhibiting promising outcomes for patients with intussusception.

## Introduction

Acute intussusception is a prevalent gastrointestinal disorder, predominantly affecting infants and toddlers. Over 95% of cases are idiopathic, without a definitive pathological cause, and manifest symptoms such as abdominal pain, vomiting, irritability, and the presence of currant jelly stool^1–3^.

Traditionally, the first-line management in our institution involves nonsurgical reduction through hydrostatic or pneumatic means, boasting an approximate success rate of 90%^4^. Despite efforts to prevent intestinal necrosis, bowel resection and loss remain unavoidable, leading to diverse opinions about the optimal strategy for intussusception among surgeons and differing institutional philosophies^5,6^.

We previously found that delayed management could contribute to a higher risk of bowel loss. The time consumed by hydrostatic or pneumatic reduction delays timely manual intervention, potentially accounting for the ultimate bowel loss. Additionally, concerns arise that excessive reduction might exacerbate the risk of intestinal ischemia, associated with intestinal necrosis. In cases presenting peritonitis, hemodynamic instability, or a deeper intussusception location—indicative of severe intestinal necrosis—timely and accurate intervention is vital. With the advancement of laparoscopic techniques in pediatric care, intussusception reduction through laparoscopy has gained traction due to its feasibility, safety, and favorable outcomes in managing intestinal necrosis^7–9^.

In 2019, we formulated an optimized management strategy for intussusception, emphasizing the laparoscopic approach. The present research aims to explore whether the liberal application of laparoscopic investigation would diminish the necessity for bowel resection through the evaluation of postoperative and long-term outcomes. This study could also serve as a foundation for future high-quality, prospective research.

## Methods

### Patients

The present study protocol was approved by the institutional review board (IRB No. 08-2023-42) of Chongqing Yongchuan Health Center for Women and Children. A retrospective analysis of patients with intussusception was conducted from April 2018 to May 2022 across three institutes, in accordance with the STROBE (Strengthening the Reporting of Observational Studies in Epidemiology) regulations. The institutional review board (IRB No. 08-2023-42) of Chongqing Yongchuan Health Center for Women and Children waived the need for written informed consent due to the retrospective nature of the study. Five attending surgeons carried out all the procedures, and pathological lead points (PLPs) causing intussusception were excluded from this research. The inclusion criteria encompassed age greater than 1 month and less than 6 years, along with the first episode and admission. Exclusion criteria included symptoms persisting for over 48 h and the presence of pathological lead points (PLPs) that were responsible for the intussusception.

### Protocol implementation

Prior to March 2020, empiric practices for the management of intussusception were commonly utilized in our institute. Patients were primarily diagnosed via sonography upon initial admission. If the duration of symptoms (DOS) was 48 h or less, an air-enema reduction was performed under a maximum pressure of 12 kPa by pediatric surgeons and radiologists. In cases where the air-enema reduction failed, an immediate exploration procedure was commenced for either surgical manual reduction or intestinal removal.

Starting in March 2020, the surgical criteria were revised to incorporate a new protocol centered around the laparoscopic approach. Briefly, the clinical management criteria under the new protocol were as follows: if the patient was in stable condition, and/or the intussusception was located in the ileocolic region as per sonograms, active air-enema reduction was conducted. Conversely, for patients displaying severe clinical status, such as persistent fever (above 38.5 °C for more than 1 days), rebounding pain, dehydrate symptom, or when the intussusception is situated in the transverse colon or beyond, intestinal necrosis was highly suspected. In these instances, liberal laparoscopic exploration and intervention were undertaken without initial hydrostatic and pneumatic reduction. Usually, during the operation, the first port was usually gone smoothly and no any injury happened in our operations. Further, we generally performed the Veress needle approach method. All patients were managed and discharged according to their individual conditions. Post-discharge, they were systematically followed up in the clinic at the end of the first week and the first month following reduction, facilitating a comprehensive documentation of symptoms related to intussusception. Ultrasonography measurements were repeated as deemed necessary, and recurrent intussusception was defined as recurrence within the first month following an initially successful reduction.

### Data involvement

For the purpose of conducting a historical control analysis concerning liberal surgical management, patients were categorized into either the pre-protocol or post-protocol cohorts based on the management protocol in use. Baseline variables-including demographic features and specific clinical characteristics—were extracted from clinical records and subsequently analyzed. Outcome characteristics encompassed both surgical and non-surgical outcomes, such as duration of surgery, blood loss, operative findings, need for resection, complication rates, admission to the intensive care unit (ICU), recurrence rate, and duration of postoperative hospital stay. The postoperative complications were ranked according to the Clavien–Dindo classification system^10^. Only grade II complications or higher, including major infections (sepsis, ventilator-associated pneumonia, and drug-resistant infections), gastrointestinal bleeding, abdominal abscess, venous thromboembolic disease, renal failure, and respiratory failures, were recorded in this research. Major complications were defined as the following situations: need for repeat laparotomy, interventional radiology procedures, or requiring admission to the intensive care unit.

The study's primary endpoints were focused on intestinal resection and recurrence, while secondary outcomes encompassed aspects like complication rates, ICU admission, duration of postoperative hospital stay, and operative duration.

### Statistical analysis

Data for the research were analyzed using SPSS version 22.0 software (SPSS Inc, Chicago, IL) and categorized into either the pre-protocol or post-protocol group based on the timeline (before or after March 2019). Univariate analysis was utilized to evaluate the comparability of the baseline data and to identify the primary and secondary endpoints. Categorical data were presented as frequencies with percentages, and were tested using Fisher's exact test for cells with frequencies less than 5; otherwise, the chi-square test was applied. Continuous data were represented as medians (interquartile ranges) or medians (ranges) for non-normally or normally distributed data, respectively, and were analyzed using the Mann–Whitney U test, the Wilcoxon rank-sum test, or Student's t-test, as appropriate. A P-value of less than 0.05 was considered indicative of statistical significance.

### Ethics approval and consent to participate

The study was approved by the institutional ethics committee at Chongqing health center for women and children, and the requirement for informed consent was waived because of the retrospective design. All methods were performed in accordance with the relevant guidelines and regulations.

## Results

### Overall characteristics

During the study period from April 2018 to May 2022, a total of 3242 consecutive patients were managed for intussusception in our institute. Sixty-three infants were excluded from the research due to unobtainable medical data, and an additional 93 cases were omitted as they were over 5 years old (n = 31) or confirmed to have pathological lead points (PLPs) (n = 62). Consequently, 3086 patients met the inclusion criteria.

In the post-protocol group (March 15, 2020, to May 1, 2022), 1748 patients were eligible for recruitment. Conversely, 1338 cases of intussusception were reviewed and recruited in the pre-protocol group (April 2018 to March 15, 2020). Of the 1338 patients reviewed during the pre-protocol period, all were treated with air reduction; 128 cases failed this treatment, resulting in a 9.6% failure rate (128/1338), with 8 cases proceeding directly to surgery due to severe complications. In total, 136 patients underwent surgical procedures, with 42 cases requiring intestinal resection.

After implementing the protocol, 1326 cases were treated with air reduction, most of which were successfully reduced, leaving only 57 failures (4.3%, 57/1326). A total of 422 cases met the criteria for immediate surgical intervention and were directly taken to the operating room for laparoscopic surgery. Of the patients managed surgically, only 39 (2.2%, 39/1748) failed manual reduction and underwent intestinal resection. The clinical variables and baseline characteristics between the two groups, including age, sex distribution, ultrasound presentation, or weight, are outlined in Table 1, with no significant differences in baseline characteristics, inflammation parameters, or ultrasonic examination.

**Table 1:** Base characteristics in children with intussusceptions management.

Characteristics	Pre protocol (n = 1338)	Post protocol (n = 1748)	P value
Male, n (%)	896 (70.0%)	1147 (65.6%)	
Female, n (%)	442 (30.0%)	601 (34.4%)	0.23
Age (months), median (IQR)	11.0 (3.2–61.6)	11.1 (4.1–60.9)	0.35
Duration of symptoms (h), median (IQR)	16 (2–48)	22 (5–48)	0.28
BMI (kg/m^2^, mean ± SD)	19.6 ± 3.1	19.8 ± 3.7	0.17
Symptoms, n (%)			
Intermittent crying	1269 (94.8%)	1653 (94.6%)	0.40
Vomiting	1132 (84.6%)	1491 (85.3%)	0.31
Bloody stool	964 (72.0%)	1247 (71.3%)	0.35
Fever > 37.5 °C, n (%)	475 (35.5%)	622 (35.6%)	0.50
Preoperative anemia (Hb < 12 g/dL), n (%)	295 (22.0)	372 (21.3%)	0.32
Leukocyte count (109/L), n (%)			
≤ 12.0	514 (38.4)	711 (40.7)	
> 12.0	824 (61.6)	1037 (59.3)	0.11
C-reactive protein (mg/L), n (%)			
≤ 8.0	420 (31.4)	556 (31.8)	
> 8.0	918 (68.6)	1192 (68.2)	0.42
PLR, mean ± SD	154.2 ± 121.2	198 ± 163.2	0.27
LCR, mean ± SD	0.13 ± 0.078	0.098 ± 0.051	0.41
Abdominal mass, n (%)	647 (48.4)	821 (47.0)	0.23
Pressure (Kp, mean ± SD)	8.94 ± 1.42	11.04 ± 1.59	0.36
Location of intussusception, n (%)			
Ileocecal valve	309 (23.1)	407 (23.3)	0.47
Ascending colon	786 (58.7)	1042 (59.6)	0.33
Transverse colon	163 (12.2)	196 (11.2)	0.22
Descending colon	59 (4.4)	71 (4.1)	0.35
Sigmoid colon	21 (1.6)	32 (1.8)	0.34

As depicted in Table 2, we conducted a comparison of the operative variables and clinical outcomes between the two groups. The interval time from initial admission to surgical intervention varied but was significantly longer in the pre-protocol group than in the post-protocol group (p = 0.008), indicating a shorter care window under the new protocol. Following the new protocol's implementation, patients exhibited fewer total intestinal resections than those in the pre-protocol group (p = 0.048, OR = 1.50; 95% CI 0.96–2.35), even though overall surgical management increased during the post-protocol period (p < 0.001, OR = 0.30; 95% CI 0.25–0.37). Interestingly, no cost increase was observed following the new protocol's implementation, as indicated by the comparison between the pre-protocol group and post-protocol group (p = 0.105).

**Table 2:** Outcomes in comparison between pre and post protocol cohorts.

	Pre protocol (n = 1338)	Post protocol (n = 1748)	p values	Odds ratio (95% CI)
Air reduction	1330 (99.4%)	1326 (75.9%)		
Direct surgical management, n (%)	8 (0.6%)	422 (24.1%)	< 0.001	0.019 (0.009–0.038)
Air reduction failed, n (%)	128 (9.6%)	57 (4.3%)	< 0.001	2.37 (1.72–3.27)
Surgical interventions, n (%)	136 (10.2%)	479 (27.4%)	< 0.001	0.30 (0.25–0.37)
Interval from initial admission to surgery, median (IQR)	9 (5–22)	6 (3–16)	0.008	
No. of patients with intestinal resection, n (%)	42 (42/1338, 3.1%)	37 (37/1748, 2.1%)	0.048	1.50 (0.96–2.35)
No. of patients with complications, n (%)	38 (38/1338, 2.8%)	47 (47/1748, 2.7%)	0.50	
No. of patients with severe complications, n (%)	17 (11/1338, 1.27%)	21 (11/1748, 1.20%)	0.50	
Hospital stay, median (IQR), days	2.1 (1.2–6.1)	2.2 (1.1–6.5)	0.37	
Recurrence, n (%)	213 (15.9%)	227 (13.0%)	0.012	1.27 (1.04–1.55)
Whole cost analysis, median (IQR), RMB, yuan	4866 (3698–17,386)	5183 (3678–18,869)	0.105	

There was a significant decrease in total recurrent events in the post-protocol group compared to the pre-protocol group (p = 0.012, OR = 1.27; 95% CI 1.04–1.55). Indeed, there are almost none recurrence following the surgical intervention for the acute intussusception. We further measured the recurrence rate exclude the cases with surgical intervention between the two groups. As indicated, there was really no difference (213/1202 vs 227/1269).

We also examined the postoperative complications within the population that underwent operative intervention based on the two protocols. As summarized in Table 3, the major postoperative complications were significantly reduced with the new protocol. Only one patient in the post-protocol group reported unplanned reoperations, compared to four patients who underwent reoperation in the pre-protocol group. This pattern signifies a trend toward more positive and effective results after the new protocol's implementation. In the post-protocol group, the Laparoscopic-open transfer and open operation rate were reduced, indicating that most of the patients could be managed laparoscopically in early stage, which should be associated with the rescue of intestinal loss. Postoperatively, there was no anastomotic leakage in the post-protocol group, whereas two patients in the pre-protocol group developed anastomotic leakage, subsequently healing with appropriate intervention. No hospital mortality occurred among the patients. Furthermore, we only compared the operated patients between the post-protocol group compared to the pre-protocol group. The result indicated no difference in term of postoperative hospital stay and overall expenses.

**Table 3:** Surgical outcomes in comparison according to the pre and post protocol.

	Pre protocol (136)	Post protocol (479)		
Types of surgery, n (%)				
Laparoscopic	122(122/136, 90.0%)	462(462/479, 96.5%)	< 0.001	0.29 (0.23–0.36)
Open	14(10.0%)	17(4.5%)	0.003	
Laparoscopic-open	21(21/122, 17.2%)	19(19/462, 4.1%)	< 0.001	
Operation duration (min, mean ± SD)	158.6 ± 47.5	169.8 ± 56.4	0.036	
Early ileus	27	35	< 0.001	3.14 (1.82–4.41)
No. of patients with intestinal resection, n (%)	42 (30.9%)	37 (7.7%)	< 0.001	5.43 (3.25–8.76)
ICU admission, n (%)	8 (7.4%)	7 (1.5%)	< 0.001	3.12 (1.59–6.50)
No. of patients with complications, n (%)	38	47	< 0.001	3.56 (2.20–5.76)
No. of patients with severe complications, n (%)	17 (17/136, 12.5%)	21 (21/479, 4.4%)	0.001	3.16 (1.59–6.09)
Shock	13	11	< 0.001	4.50 (1.97–10.28)
Pneumonia	19	22	< 0.001	3.37 (1.77–6.44)
SSI	22	31	0.001	2.79 (1.56–5.00)
Unplanned reoperations	4	1	0.01	14.48 (1.61–130.70)
Postoperative hospital stay (days, mean ± SD)	5.2 ± 3.7	3.3 ± 2.5	0.014	
Cost analysis for patients operated, median (IQR), RMB, yuan	10,698 (8851–17,897)	9967 (7983–16,537)	0.126	

## Discussion

In developing countries, bowel loss among children is predominantly linked to the incidence of intussusception. In this study, we identified that a strategy incorporating more liberal laparoscopic interventions led to favorable outcomes in terms of intestinal resection and recurrence, as opposed to those observed in the pre-protocol period. This discovery underscores the potential relevance of the new management approach in reducing intestinal loss and optimizing patient outcomes.

Timely reduction during the obstruction of bowel blood flow is vital for rescuing strangulated intussusception and consequently minimizing eventual bowel resection^11,12^. Traditionally, pneumatic enema reduction at maximum pressures of 100 mm Hg has been the first-line treatment for intussusception, boasting an overall success rate of up to 90%^13^. During our pre-protocol period, the majority of patients were successfully managed through pneumatic reduction. However, surgical intervention was essential for approximately one-tenth of all intussusception admissions, due to distinct contraindications for pneumatic reduction or failure of the procedure^14^. Among those who required surgery, 34% needed intestinal resection, even after an initial attempt at air reduction. Intestinal resection typically arises as a grave complication in cases of intussusception necrosis.

The procedure of pneumatic enema reduction, which may take several hours and apply high pressure to the affected ischemic bowel, thereby compromising blood supply, could contribute to the necrosis of the intussusception. Thus, it may increase the likelihood of intestinal resection. The core rationale behind the current protocol is to avoid excessive waiting times for failed air reduction—a hazardous situation that permits a second exposure to anesthesia and surgical intervention. The decrease in intestinal resection and the increase in surgical reduction was caused by the liberal surgical intervention. At our institute, intussusception management has evolved from active air reduction to early operative action with the adoption of the laparoscopic approach. The delayed repeat enemas (DREs) was reported as the enemas repeated after an initial failed enema^15^. Indeed, the success rate is low with extremely painful experiences for the patients^16^. In China, including our institute, this strategy is seldom adopted.

The adverse impact of therapeutic pneumatic reduction in severe cases is illuminated in the current series, where increased intestinal resection and surgical complications were observed. A delayed response to bowel ischemia may lead to unnecessary bowel resection and loss, constituting grave complications in intussusception management^17^. In such circumstances, precise laparoscopic exploration is favored for patients with severe incarceration to rescue the ischemic intussusception and minimize signs of perforation, thereby preventing sepsis development as swiftly as possible. Based on our experience and previously identified risk factors, air reduction was only applied to patients with ileocolic intussusception presenting symptoms within a 48-h window. Other studies have linked the duration of onset of intussusception symptoms to the likelihood of bowel loss^18,19^, a finding congruent with our observation that timely laparoscopic intervention could curtail intestinal resection.

Laparoscopic reduction of idiopathic intussusception was first documented in 1996^20^. Since that time, liberal laparoscopic procedures have been increasingly adopted in pediatric intussusception cases, with rare conversion^21^. We initiated laparoscopic intussusception reduction in 2016, making it our standard practice. We have shown that current interventional laparoscopic techniques have proven valuable in handling patients with severe symptoms, including signs of perforation and peritonitis. Using the laparoscopic view, the surgeon can employ atraumatic bowel graspers with continuous gentle pulling to restore the intussusception, and easily inspect for perforation, necrosis, and lead points once reduction is achieved. The decrease in postoperative complication and postoperative hospital stay was due to the liberal laparoscopic surgery. Our learning curve may also affect this result. The majority of intussusception cases recover without complications under laparoscopic care. Here, we need to archive a 1% decrease in intestinal resection with the cost of a 17% increase in surgical treatment. most pediatric surgeons would argue that Surgical intervention itself is one of the most severe harms and should avoided as much as possible in the treatment of pediatric intussusception. Indeed, the laparoscopic management has the advantages of minimally invasive and fast recovery and can be safely and effectively executed in most patients, which favor the liberal surgical laparoscopy reduction, albeit not without occasional failure. Given these advantages, we favor laparoscopic exploration for intussusception management, as surgical complications are rarely encountered following this intervention.

The chief concern in intussusception management is bowel loss. During the pre-protocol period, high-pressure reduction might lead to intestinal resection due to bowel ischemia. Beyond the initial 48–72 h, air reduction of intussusception carries a substantial risk of failure and subsequent bowel loss. Timely manual reduction via laparoscopic exploration, as opposed to air reduction, affords a reasonable timeframe to forestall further ischemic damage. Encouragingly, our post-protocol cohort witnessed a reduction in the number of patients requiring intestinal resection, marking an improvement over the pre-protocol cohort and other reported cases.

During the follow-up research, we observed a low recurrence incidence, consistent with previously reported rates^22^. It can be inferred that the higher number of surgical interventions may contribute to this low recurrence rate. Additionally, the relatively short follow-up period might also partially account for the low incidence. Although care costs were not evaluated in this study, increased surgical management in our post-protocol care could be correlated with higher care expenses, which may offset the benefits of reduced intestinal resection and recurrence.

Several limitations must be taken into account when interpreting the results. Firstly, this research constitutes a retrospective review, potentially diminishing the reliability of the findings. Given the relatively low incidence of bowel loss, there appears to be an insufficient number of cases to detect any significant differences. A randomized controlled trial would be more illuminating concerning the nature of intestinal resection. Decision-making regarding surgical intervention, intestinal necrosis evaluation, and resection must rely on the individual judgment of the attending surgeon, thus rendering the conclusion about the efficacy of this new strategy non-arbitrary. To specifically validate the advantages of the new protocol in patients with intussusception, further comprehensive multicenter collaborative research should be pursued.

## Conclusion

In conclusion, bowel loss continues to be a primary concern in the management of intussusception. Individual judgment regarding surgical intervention, intestinal necrosis evaluation, and resection must be taken seriously. An emphasis on early referral to specialized pediatric surgical centers for surgical intervention evaluation in patients with intussusception is warranted.

## Data Availability

The dataset analyzed during the current study are available from the corresponding author on reasonable request.
